# Two Functional Classes of Rod Bipolar Cells in the Healthy and Degenerated Optogenetically Treated Murine Retina

**DOI:** 10.3389/fncel.2021.809531

**Published:** 2022-01-13

**Authors:** Giulia Schilardi, Sonja Kleinlogel

**Affiliations:** Institute of Physiology and Department of Biomedical Research (DBMR), University of Bern, Bern, Switzerland

**Keywords:** rod bipolar cell, retinal degeneration, BK channels, vision restoration, electrophysiology, optogenetics

## Abstract

Bipolar cells have become successful targets for optogenetic gene therapies that restore vision after photoreceptor degeneration. However, degeneration was shown to cause changes in neuronal connectivity and protein expression, which may impact the quality of synthetically restored signaling. Further, the expression of an optogenetic protein may alter passive membrane properties of bipolar cells affecting signal propagation. We here investigated the passive membrane properties of rod bipolar cells in three different systems, the healthy retina, the degenerated retina, and the degenerated retina expressing the optogenetic actuator *Opto-mGluR6*. We found that, based on the shape of their current-voltage relations, rod bipolar cells in healthy and degenerated retinas form two clear functional groups (type 1 and type 2 cells). Depolarizing the membrane potential changed recorded current-voltage curves from type 1 to type 2, confirming a single cell identity with two functional states. Expression of *Opto-mGluR6* did not alter the passive properties of the rod bipolar cell. With progressing degeneration, dominant outward rectifying currents recorded in type 2 rod bipolar cells decreased significantly. We demonstrate that this is caused by a downregulation of BK channel expression in the degenerated retina. Since this BK conductance will normally recover the membrane potential after RBCs are excited by open TRPM1 channels, a loss in BK will decrease high-pass filtering at the rod bipolar cell level. A better understanding of the changes of bipolar cell physiology during retinal degeneration may pave the way to optimize future treatment strategies of blindness.

## Introduction

Bipolar cells (BCs) are the first-order interneurons of the retina receiving direct input from the light-sensitive photoreceptors. BCs are divided into ON-type BCs (OBCs) and OFF-type BCs. The OBCs are, in turn, divided into cone and rod BCs (RBCs) based on the photoreceptor connectivity (Lorber et al., 2016; Martemyanov and Sampath, 2017). Amacrine, horizontal, and ganglion cells (RGCs) form close contacts with BCs and together they create the diverse receptive fields of RGCs, which transmit the visual information to the brain (Nelson and Kolb, 1985; Vries et al., 2011; Chaya et al., 2017).

Genetic mutations in photoreceptors and retinal pigment epithelial cells, but also oxidative stress and other factors can lead to photoreceptor death, with a high primary prevalence for rods (Hartong et al., 2006; Mustafi et al., 2016; Newton and Megaw, 2020). Degeneration of rods leads to night-blindness, an early symptom of patients suffering from retinitis pigmentosa, followed by secondary cone loss and eventual blindness (Ferrari et al., 2011; Mustafi et al., 2016). Despite the loss of photoreceptors, inner retinal neurons survive for years (Strettoi and Pignatelli, 2000; Chang et al., 2002). Indeed, a recent expression study found no changes in expression levels of proteins involved in cell survival, neuronal growth, and calcium homeostasis in OBCs of the degenerated retina (Gilhooley et al., 2021). This renders OBCs promising targets for optogenetic vision restoration. Since BCs are the first-order interneurons of the retina, light-activation of BCs could conserve parallel information processing within the retina and potentially restore higher quality vision compared to optogenetically targeted RGCs (Sahel et al., 2021). The inhibitory inner retinal circuitry separates transient and sustained responses, responses at light onset or light offset, and computes complex features like direction selectivity and edge detection (van Wyk et al., 2015a). Channelrhodopsin-2 (Lagali et al., 2008), rhodopsin (Cehajic-Kapetanovic et al., 2015) middle-wave cone opsin, and Opto-mGluR6 (van Wyk et al., 2015a) have been introduced into OBCs of blind retinal degeneration (*rd1*) mice and were shown to restore light sensitivity and basic levels of visual processing. Recently developed synthetic promoters enabled specific and efficient expression of *optogenes* in the OBCs, supporting future clinical applications of OBC-targeted optogenetics (Hulliger et al., 2020). However, although OBC targeted optogenetic interventions were successfully employed in *rd1* mice, it is known that retinal degeneration causes neuronal rewiring, changes in gene expression, and protein re-localization (Strettoi et al., 2002; Marc et al., 2007; Soto and Kerschensteiner, 2015) as well as neurochemical remodeling (Chua et al., 2009). These modifications may affect the quality of synthetically restored signaling, which has never been investigated.

RBCs represent the majority of OBCs and convey scotopic (low light intensity) vision as opposed to photopic vision carried primarily by the cone system (Behrens et al., 2016). Fifteen different cone-bipolar cell types are described, but only one morphological type of RBC (Shekhar et al., 2016). RBCs, as opposed to cone bipolar cells, do not directly contact RGCs, but they hijack the cone system by feeding their signal into the AII amacrine cells *via* an excitatory (glutamatergic) synapse (Wässle, 2004; Lorber et al., 2016). RBCs express an inventory of ion channels, including calcium, HCN, and potassium channels (Hook et al., 2019), and, apart from the AII cells, also make glutamatergic synapses onto A17 amacrine cell processes that project back onto RBC terminals forming inhibitory (GABAergic) synapses (Euler and Wässle, 1995; Hartveit, 1999). The ensemble of functional channels determines the resting membrane potential and reactivity of the RBC. In the healthy retina, the resting membrane potential of the RBC lies at approximately −45 mV (Euler and Masland, 2000; Pang et al., 2004). Some studies reported depolarized OBCs in the degenerating retina causing increased excitability of the cone OBC-amacrine cell network underlying oscillatory activity in the *rd1* retina (Trenholm et al., 2012; Choi et al., 2014; Menzler et al., 2014). Other studies have shown that OBCs in the *rd1* retina become more hyperpolarized (Borowska et al., 2011; Laprell et al., 2017).

To better understand RBC physiology in health and degeneration we set out to characterize the ion channel inventory and the changes in RBC membrane physiology in wild-type and *rd1* retinas expressing the optogenetic designer protein Opto-mGluR6 (van Wyk et al., 2015a).

## Materials and Methods

### Animals and Tissue Preparation

Male and female (p175) *C57BL/6J (N = 10), C57BL/6J_Opto-mGluR6 (N = 10)*, and *FVB/NCrl_Opto-mGluR6* mice (p175, *N* = 15) were used for retinal patch-clamp recordings. All animal experiments were conducted in accordance with the Swiss Federal Animal Protection Act for the care and use of animals and the standards set forth in the ARVO Statement for the Use of Animals in Ophthalmic and Visual Research and were approved by the animal research committee of Bern. *FVB/NCrl* mice are homozygous for the retinal degeneration 1 mutation (*Pde6b*), causing primarily rod photoreceptors to degenerate and leading to blindness by weaning age (Han et al., 2013). The same mutation causes autosomal retinitis pigmentosa in human patients. *FVB/NCrl_Opto-mGluR6* and *C57BL/6J_Opto-mGluR6* are transgenic lines on *rd1* or wild-type background, respectively, expressing the Opto-mGluR6 *optogene* and a red fluorescent marker (TurboFP635) selectively in the retinal ON-bipolar cells (van Wyk et al., 2015a, b).

Mice were dark-adapted for 1 h prior to euthanasia using cervical dislocation. The retina was rapidly removed under far-red light to avoid bleaching of the photoreceptors in C57BL/6*J* retinas. Retinas of *C57BL/6J* and * C57BL/6J_Opto-mGluR6* mice were embedded in 1% Agarose (Sigma) and sectioned (250 μm) using a Leica 1000s Vibratome (Arman and Sampath, 2010). The slices were kept at 4°C on ice in oxygenated Ames’s medium (95% O_2_). Since *FVB/NCrl_Opto-mGluR6* mice lack the photoreceptor layer, patching was performed in whole-mount retinas attached to a coverslip (Walston et al., 2018).

### Whole-Cell Patch-Clamp

#### RBC Recordings

Recordings were performed in darkness at 34°C with an Axopatch 200B amplifier (Axon instruments). The recorded signals were low-pass filtered at 2 kHz. Retinal slices and whole-mounts were perfused at a flow rate of 5 ml/min with gassed Ames’s medium (95% O_2_ and 5% CO_2_). Patch pipettes (Harvard apparatus) were pulled at a resistance of 8–12 MΩ using a ZEIT DMZ puller (Germany). Seal resistances ≥2 GΩ were commonly obtained and monitored for consistency. Series resistance (RS) was continuously monitored and leak currents up to 100 pA were accepted. Only one cell was recorded from each retinal slice/whole-mount. Whole-cell voltage ramp protocols (from −70 mv to +140 mv, 2 s) from the resting membrane potential (E_m_) were used to study voltage-current (I/V) relationships and current steps (−10 pA to +90 pA, Δ10 pA), applied to evaluate the BK channel involvement in the control condition and in the presence of the specific BK channel agonist (NS1619; 30 μM) and antagonist (Paxilline; 5 μM; Yamamura et al., 2001; Abdullaev et al., 2010; Zhou and Lingle, 2014). The same RBC has been tested in the three different conditions. TPMPA 50 μM and SR955 31 10 μM were used to block GABA-C and GABA-A receptors, respectively, on RBCs dendrites (George and Sadiq, 2021).

#### Solution and Drugs

The slicing solution was Ames’ medium (Sigma) containing 2.38g/L HEPES and 0.887g/L sodium chloride (7.4 pH, 280 mOsm). As an external solution for whole-cell recordings and for retinal dissections, we used sodium hydrogen carbonate (NaHCO3) buffered Ames’s medium. The pipette solution contained 100 mM potassium chloride (KCl), 1 mM sodium chloride (NaCl), 2 mM HEPES, 0.1 mM guanosine triphosphate (GTP), 1 mM adenosine triphosphate (ATP), 0.1 mM cyclic guanosine monophosphate (cGMP), 1.5 mM cyclic adenosine monophosphate (cAMP), 5 mM ethylene glycol-bis(2-aminoethylether)-N,N,N’,N’-tetraacetic acid (EGTA), 0.1 mM magnesium chloride (MgCl_2_) and 0.5 mM calcium chloride (Cl_2_, pH 7.4). Stock solutions of agonists and antagonists were prepared in dimethyl sulfoxide (DMSO) and stored at −20°C. Pharmacological agents were dissolved in AMES medium to the working concentration on the day of the experiment and washed in and out for 2 min each, allowing for full solution exchange in the recording chamber. Drugs were applied sequentially and drug effects were fully reversible with washout (Supplementary Figure 1).

#### Cell Type Identification

In retinal slices, RBC somas were identified with an upright microscope (Nikon Eclipse E600FN) equipped with an infrared GP-CAM3 Altair Astro camera, differential interference contrast (DIC) optics, and epifluorescence illumination. 0.03% Lucifer yellow potassium salt (Sigma Aldrich) or 2% biocytin (Sigma Aldrich) was added to the intracellular solution to label recorded cells. Lucifer yellow-labeled cells were visualized live during the electrophysiological recording whereas whole-mounts containing biocytin labeled cells were fixed with 4% PFA for 15 min for subsequent immunohistochemical analysis. Light-induced voltage responses were recorded in a subset of cells to confirm typical light-activated RBC responses (Euler and Masland, 2000; Pang et al., 2004). The two-second long blue (500 nm) light stimulus was generated with a pE-4000 epi-fluorescence light source from CoolLED. The light intensity was reduced to 9.6 × 10^9^ photons cm^−2^ s^−1^ using ND filters, measured with a PM200 light meter (Thorlabs). In the retinal whole-mount of the *rd1* retina, RBCs were exposed at the surface of the retinsa. In the *rd1*_Opto-mGluR6 retina, OBCs were identified *via* the red TruboFP635 fluorescence.

### FACS Sorting and qPCR

*C57BL/6J_Opto-mGluR6* mice (p175) were used as control, as their retina presents healthy tissue with all the retinal layers present. *FVB/NCrl_Opto-mGluR6* mice and *C3H/HeOuJ_Opto-mGluR6* at p70 and p175 were used as models of retinal degeneration (van Wyk et al., 2015a, b). Fourteen retinas from seven mice were pooled. After retinal dissociation, OBCs were sorted by TurboFP635 fluorescence with 68.15 ± 3.65% positive living cells extracted for *C57BL/6J_Opto-mGluR6*, 55.1 ± 1.1% for *FVB/NCrl Opto-mGluR6* and 48.5 ± 1.2% in *C3H/HeOuJ_Opto-mGluR6 retinas*. The extraction of RNA was performed using the SV Total RNA Isolation Kit (Promega, Dübendorf, Switzerland) and one-step quantitative reverse-transcription PCR (qPCR) reactions (40 cycles) using the KAPA SYBR FAST One-Step Universal Kit (Kapa Biosystems, London, UK) on an Eco Real-Time PCR System (Illumina Inc., San Diego, CA). Values obtained for BK channel RNA (F 5’-CTGCAGGCGGCTGATCTATT-3’, R 5’-TCATGCCTCATCAGCTTCGG-3’) were normalized against the RNA levels of RPL8, which was selected as a housekeeping gene since its expression was shown to remain stable during retinal degeneration (Hulliger et al., 2020).

### Immunohistochemistry

Immunolabeling of cryofixed vertical retina sections of *C57BL/6J_Opto-mGluR6 mice* (p175) and *FVB/NCrl_Opto-mGluR6* mice (p70) and (p175) was performed using standard protocols with primary antibodies against BK channels (1:100, Alomone Labs Cat# APC-15, RRID: AB_2313725), protein kinase C (1:750, Invitrogen Cat#sc8393, RRID: AB_628142; Xiong et al., 2015) and TRPM1 (1:100, BiCell Scientific Cat# 11021, RRID: AB_2895222) and secondary polyclonal antibodies against Alexa 488 (1:400, Invitrogen Cat# A11008, RRID: AB_143165 and Cat# A11006, RRID: AB_2534074) and CY3 (1:400, Invitrogen Cat#A10521, RRID: AB_2534030). Immunolabeling for the morphological identification of RBCs in *FVB/NCrl_Opto-mGluR6* retinal whole-mounts was performed with Streptavidin-Alexa 488 to label biocytin-injected cells (1:400, Invitrogen Cat#S11223). Images were taken using either a Zeiss LSM880confocal microscope with a 63× objective (NA: 0.8) or a Zeiss Axio Vert.A1 epifluorescence microscope with a 40× objective (NA:0.6).

### Data Analysis and Statistics

Electrophysiological data were analyzed with pCLAMP 10.7 (Molecular devices) and Prism 5 (GraphPad Software, San Diego, CA, USA). RBCs have been categorized in type 1 and type 2 cells according to the fitting of the I/V curve with the same polynomial equation (see below). Wolfram Alpha software has been used to solve the polynomial equation and calculate the inversion membrane potential (Erev). The I/V relation for the type 2, for *C57BL/6J, C57BL/6J_Opto-mGluR6*, and *FVB/NCrl_Opto-mGluR6* conditions has been fitted with a linear equation to evaluate deviation from linearity and the relative membrane potential at which this occurs (Erec). The Shapiro test has been performed to evaluate the Gaussian distribution of the data. When the data were equally distributed we used the parametric Student’s t-test, when data were not equally distributed we used the non-parametric Mann-Whitney test. In the figures, a single asterisk (*) indicates a *P* value of *p* ≤ 0.05, (**) indicates a *P* value of *p* ≤ 0.02, (***) indicates a *P* value of *p* ≤ 0.001. Results are presented as average values ± standard error (SEM).


$$y=ax^{6}+bx^{5}+cx^{4}+dx^{3}+ex^{2}+f+g$$

*type* 1(C57BL/6J,FVB/NCrl_Opto)


$$y=ax^{5}+bx^{4}+cx^{3}+dx^{2}+ex+f$$

*type* 2* (C57BL/6J)


$$y=ax^{3}+bx^{2}+cx+d$$

*type* 2* (FVB/Crl_rd1_Opto)

The data from the acquired FACS files were *analyzed* using FlowJo software (FlowJo LLC, Ashland, OR, United States). All samples were gated using the same gating tree and gate positions: (1) forward scatter area (FSC-A) vs. side scatter area (SSC-A) to gate ON-Bipolar cells; (2) FSC-A vs. FSC-H to gate for single cells and exclude cell aggregates or multiple cells measured simultaneously; (3) SYTO16 vs. PI to gate for their fluorescent signal intensities. Data are displayed as pseudocolor density scatter plots (Herzenberg et al., 2006). An untreated control sample was used as a reference in order to set the position of gate R1. The other gates were set based on histograms showing positions of optimal separation of two neighboring groups.

## Results

### The Membrane Potential as a Toggle Switch for Rod Bipolar Cell (RBC) Function

**Figure 1:**
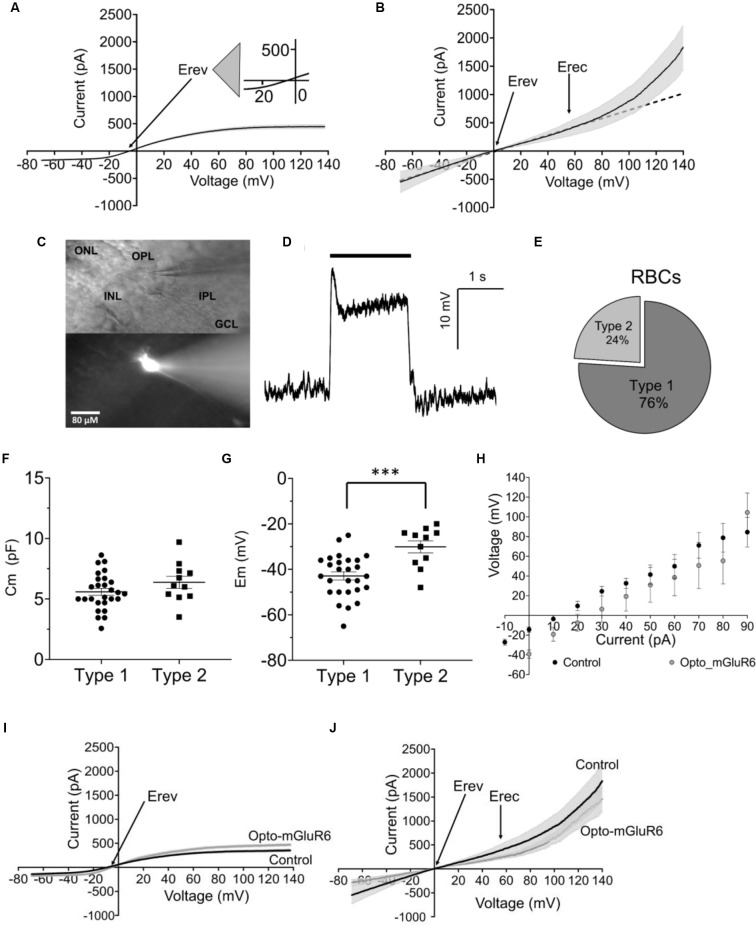
Functional rod bipolar cell types in the *C57BL/6J mouse* retina. **(A)** I/V relationship (ramp stimulus) for type 1 (*n* = 35). **(B)** I/V relationship (ramp stimulus) for type 2 (*n* = 11), line equation y = ax+b, *s.e.m*. in **(A,B)** shown as gray underlay, Erev reversal potential, Erec rectification potential. **(C)** DIC (top) and epifluorescent (bottom) example micrograph of a retinal slice with a Lucifer Yellow injected RBC, visualized live during the electrophysiological recording by a Nikon Eclipse E600FN (40×, NA: 0.80) microscope equipped with an infrared GP-CAM3 Altair astro camera. **(D)** Example light-response of a current-clamped RBCs to a 2 s light stimulus (9.6 × 109 photons cm^−2^ s^−1^, 500 nm). **(E)** Relative fractions of type 1 and 2 RBCs. **(F)** Average cell membrane capacitance (Cm) for type 1 (*n* = 27) and type 2 (*n* = 11) RBCs. **(G)** Average resting membrane potentials (Em) for type 1 (*n* = 27) and type 2 RBCs (*n* = 11), ***p = 0.001. **(H)** Average values of membrane potential in response to current injection steps for RBCs *C57BL/6J* retinas (control, *n* = 21) and RBCs from *C57BL/6J_Opto-mGluR6* retinas (Opto-mGluR6, *n* = 5). **(I)** I/V relationship (ramp stimulus) of type 1 RBCs from *C57BL/6J* retinas (*n* = 21), and *C57BL/6J_Opto-mGluR6* retinas (*n* = 5). **(J)** I/V relationship (ramp stimulus) of type 2 RBCs from *C57BL/6J* retinas (*n* = 11) and *C57BL/6J_Opto-mGluR6* retinas (*n* = 3).

We recorded voltage-clamp ramp protocols (−70, +140 mV) from RBCs in retinal slices of wild-type C57BL/6*J* mice and analyzed current-voltage (I/V) relationships. By fitting the I/V curves with polynomial equations (see Data Analysis, Material and Methods), we identified two functionally different RBC types (Figures 1A,B). Type 1 had a significantly more hyperpolarized reversal potential (Erev = −5.28 ± 0.56 mV, *p* ≤ 0.02) and a belly-shaped I/V relationship with an outward and an inward current (Figure 1A). Type 2 RBCs had a reversal potential close to 0 mV (Erev = −0.54 ± 1.28 mV) and a strong outward rectifying current at depolarized potentials above 48.78 ± 16.26 mV (Erec; Figure 1B). Using Lucifer yellow (0.03%) in the pipette solution confirmed that both response types shared the same morphology (Figure 1C). The position of the soma in the proximal inner nuclear layer (INL) in close contact with the photoreceptors, the position of the axon terminal in layer 4–5 of the inner plexiform layer (IPL), and the clustered dendrites clearly identified both, type 1 and type 2 functional types as of the same anatomical RBC class (Euler and Masland, 2000; Pang et al., 2004). We additionally probed RBC identity by whole-cell current clamp recordings activating RBCs with a 2-second long scotopic light stimulus (9.6 × 10^9^ photons cm^−2^ s^−1^, 500 nm; Figure 1D). Light responses were typical for RBCs and characterized by a strong transient initial depolarization followed by a sustained response component and a hyperpolarization at light offset (Euler and Masland, 2000; Pang et al., 2004). We found more functional type 1 (76%) than type 2 (24%) RBCs (Figure 1E). Type 1 and type 2 RBCs had similar membrane capacitances (type 1, Cm: 5.51 ± 0.21 pF; type 2, Cm: 5.05 ± 1.39 pF; Figure 1F) again corroborating that they were of the same anatomical cell type. However, type 1 and type 2 RBCs differed clearly in their resting membrane potentials (Em; type 1: −43.48 ± 1.44 mV; type 2: −30.09 ± 2.78 mV), with type 2 being significantly more depolarized (Figure 1G; *p* = 0.0007). Consequently, the Em seems to trigger the activity of different ion channels. Importantly, expression of Opto-mGluR6 had no effect on RBC passive physiology, such as the membrane potential (Figure 1H), Erev (type 1: −5.69 ± 0.91 mV, type 2: −4.4 ± 1.2 mV), total currents [I(tot)140 mV: type *1* = 473.9 ± 211.9 pA, type *2* = 1457.0 ± 252.9 mV] or the I/V relationship (Figures 1I,J).

**Figure 2:**
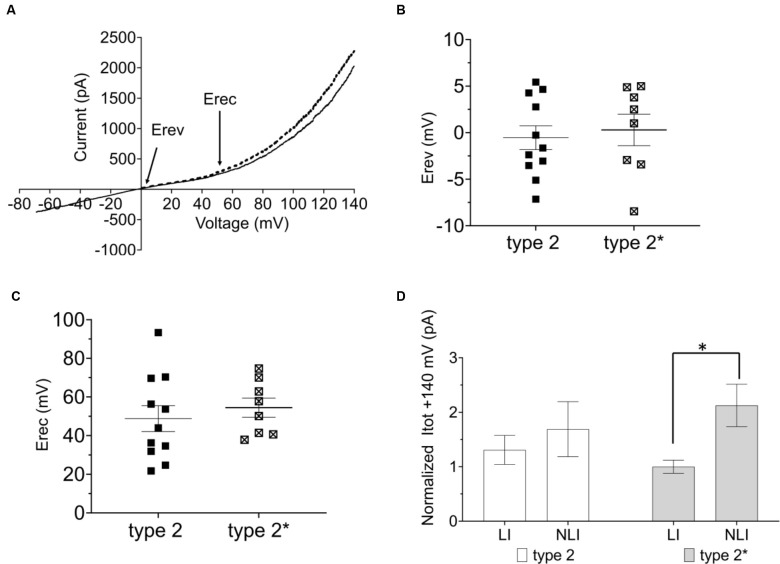
The resting membrane potential as toggle switch for the functional RBC type in the *C57BL/6J mouse*. **(A)** I/V relationship (ramp stimulus) for type 2 (*n* = 11, solid line) and 2* (*n* = 8, broken line). **(B)** Average values of reversal potential (Erev) for native type 2 RBCs (*n* = 11) and converted type 1 RBCs by positive current injection (type 2*; *n* = 8). **(C)** Average values of rectification potential (Erec) for native type 2 RBCs (*n* = 9) and 2* RBCs (*n* = 8). **(D)** Average values of linear (LI) and non-linear (NLI) current components of the total current at +140 mV (ltot) for type 2 (*n* = 9) and 2* RBCs (*n* = 8), *p = 0.03.

We next tested if the two functional RBC types were interconvertible by manipulation of Em. For this, we injected positive current into type 1 RBCs of *C57BL/6J* retinal slices to depolarize the membrane potential to that of type 2 RBCs and again characterized the I/V relationship. Indeed, the type 1 RBC was converted to a type 2 RBC electrophysiological response, with a typical strong outward rectifying current (Figure 2A). When comparing Erev and Erec we did not find any difference between the converted type 1 (type 2*, Erev 0.30 ± 1.70 mV, Erec: 54.44 ± 4.99 mV) and the original type 2 RBC (type 2, Erev: −0.54 ± 1.28 mV, 48.79 ± 6.70 mV; Figures 2B,C). We calculated the linear (LI) and non-linear (NLI) current components of the total current at +140 mV (Itot). LI and NLI were identical in the converted type 2* (LI: 717.97 ± 86.39 pA; NLI 1525.66 ± 279.51 pA) and the original type 2 RBC (939.26 ± 191.47 pA; NLI 1212.75 ± 362.29; Figure 2D). However, in type 2* RBCs the NLI was statistically larger than LI (*p* = 0.03), which was not the case in original type 2 RBCs (Figure 2D). Again, *Opto-mGluR6* expression had no effect on Erec (59.96 ± 8.86 mV) or the proportions of LI (597. 28 ± 111.54 pA) and NLI (859. 72 ± 222.76 pA) current components of type 2 RBCs.

These results together confirm that the resting membrane potential manipulates the passive membrane properties of RBCs, and consequently the RBC’s response to the application of the same ramp voltage protocol. Expression of Opto-mGluR6 had no effect on the passive membrane properties of RBCs.

### Characterization of Rod Bipolar Cells in the Degenerated and Optogenetically Treated *rd1* Retina

**Figure 3:**
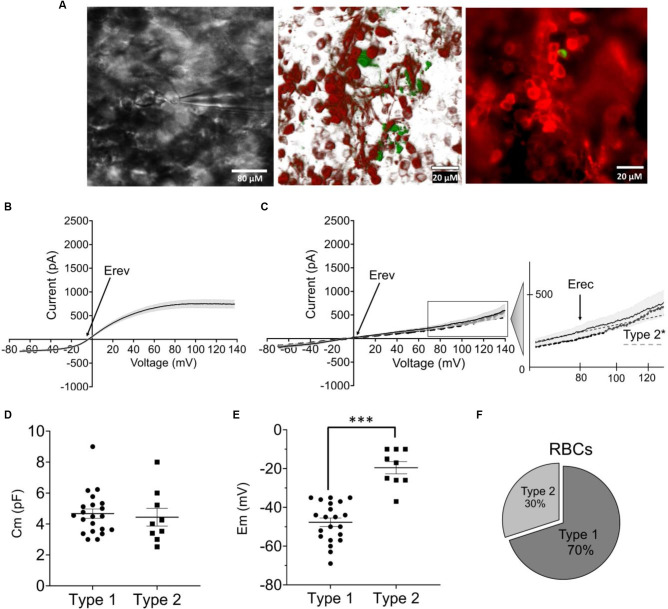
Functional characterization of RBCs in *the* optogenetically treated *rdl* mouse retina *(FVB/NCrl_ Opto-mGluR6* mouse model). **(A)** Left: live image of *a* RBC during electrophysiological recording visualized by a Nikon Eclipse E600FN (40×, NA: 0.80) microscope equipped with an infrared GP-CAM3 Altair astro camera; middle: confocal reconstruction of labeled RBC. Image was taken on a Zeiss LSM880 confocal microscop e (63×, NA: 1.4) of eight optical sections (tot: 224 μM with step-size of 28 μM); right: photomicrograph of an injected and immunohistochemically labeled RBC (green) as one of the TurboFP635 expressing OBCs taken on a Zeiss Axio Vert A1 epifluorescence microscope (40×, NA: 0.6). **(B)** I/V relationship (ramp stimulus) for type 1 RBCs (*n* = 21). **(C)** I/V relationship (ramp stimulus) for native type 2 (continous line, *n* = 9) and converted 2* *RBCs* (*dotted line*, *n* = 11), line equation y = ax+b. *s.e.m*. in **(B,C)** shown as gray underlay, Erev reversal potential, Erec rectification potential. **(D)** Average membrane capacitance (Cm) for type 1 (*n* = 21) and type 2 (*n* = 9) RBCs. **(E)** Average resting membrane potentials (Em) for type 1 (*n* = 21) and type 2 RBCs (*n* = 9), ***p = 0.001. **(F)** Relative fractions of type 1 and 2 RBCs across recordings.

We again recorded I/V relationships, this time from RBCs in retinal whole-mounts of fully degenerated transgenic *FVB/NCrl_Opto-mGluR6 mice*. RBCs were positively identified by the expression of the red fluorescent protein TurboFP635 and their direct accessibility to the patch pipette from the degenerated photoreceptor side. Recorded cells were labeled with biocytin contained in the pipette for subsequent anatomical analysis (Figure 3A). Also in the *rd1* retina, we identified two functionally different RBC types that were of the same anatomical cell type (Figures 3B,C). As in the *C57BL/6J* retina, both functional RBC types possessed similar Cm (Figure 3D type 1 Cm = 4.66 ± 0.42 pF; type 2 Cm = 4.44 ± 0.57 pF), with type 2 RBCs having a more depolarized Em (−19.55 ± 3.15 mV) in comparison to type 1 RBCs (−47.71 ± 2.70 mV, *p* = 0.001; Figure 3E). Also in the degenerated retina, type 1 RBCs were more abundant (70% of cells recorded) compared to type 2 RBCs (Figure 3F).

**Figure 4:**
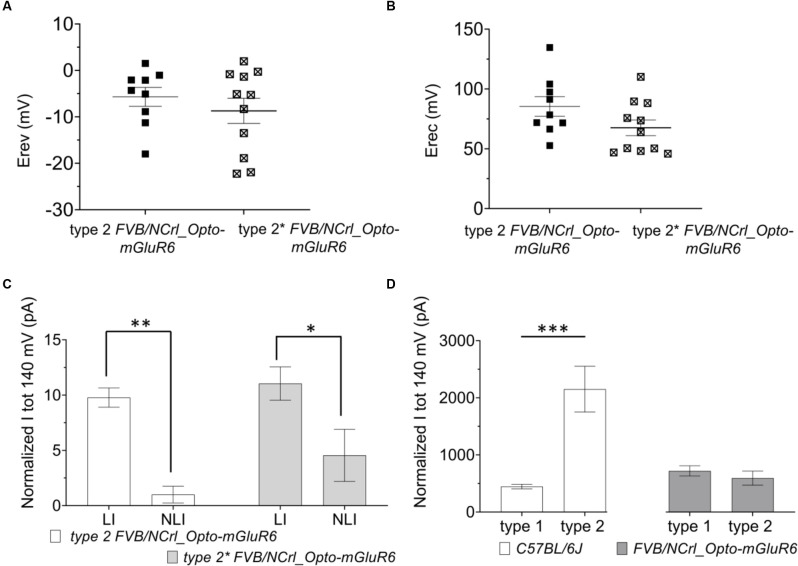
The resting membrane potential of RBCs determines the functional state (type 1 or type 2) in the *FVB/NCrl_Opto-mGluR6* mouse. **(A)** Averages values of reversal membrane potential (Erev; mV) for type 2 *FVB/NCrl_Opto-mGluR6* RBCs (*n* = 9) and type 2* (*n* = 11). **(B)** Average values of rectification potential (Erec, mV) for type 2 *FVB/NCrl_Opto-mGluR6* RBCs (*n* = 9) and type 2* (*n* = 11). **(C)** Average values of linear (LI) and non-linear current (NLI) current components of the total current at +140 mV (ltot, pA) for type 2 *FVB/NCrl_Opto -mGluR6* (*n* = 9) and type 2* (*n* = 9) **p (LI/NLI type 2) = 0.01, *p (LI/NLI type 2*) = 0.03. **(D)** Averages values of total current at +140 mV (ltot; pA) for type 1 and type 2 in *C56BL/6J* and *FVB/NCrl_Opto-mGluR6*, ***p (typel/type2 C57BL6/6J) ≤ 0.0001.

Again, type 1 RBCs could be interconverted into type 2 RBC by positive current injection depolarizing the resting membrane potential (Figure 3C: type 2*). Again, we found no significant differences between Erev (Figure 4A: type 1: −5.68 ± 2.02 mV; type 2*: −8.01 ± 2.54 mV) and Erec (Figure 4B: type 1 85.41 ± 8.01 mV; type 2* 67.53 ± 4.79 mV) in the native and created type 2 RBCs. The total current at +140 mV was similar in type 2 RBCs (593.87 ± 123.63 pA) and type 2* RBCs (410.53 ± 67.77 pA), but the distribution of LI and NLI were very different in the *rd1* retina compared to the C57BL/6 retina. LI had a significantly bigger contribution to the total current in the *rd1* retina in both, type 2 RBCs (type 2 LI: 420.60 ± 58.45 pA; type 2 NLI: 173.26 ± 90.20 pA, *p* = 0.01) and type 2* RBCs (type 2* LI: 372.44 ± 36.64 pA; type 2* NLI: 38.09 ± 58.40 pA, *p* = 0.03; Figure 4C). Compared to RBCs in the *C57BL/6J* retina, type 1 RBCs were more hyperpolarized (type 1 C57BL/6J: −43.48 ± 1.44 mV; type 1 *FVB/NCrl_Opto-mGluR6*: −47.71 ± 2.70 mV) and type 2 RBCs more depolarized (type 2 *C57BL/6J*: −30.09 ± 2.78 mV, type 2 *FVB/NCrl_Opto-mGluR6*: −19.55 ± 3.15 mV, *p* = 0.05). Interestingly, the total current of type 1 RBCs (720.15 ± 85.18 pA) was doubled compared to the *C57BL/6J* retina (445.89 ± 39.96 pA), whereas type 2 currents were significantly reduced in the *rd1* retina (593.87 ± 123.63 pA) to about a quarter of those seen in *C57BL/6J* retinas (2,151.67 ± 261.91 pA; *p* ≤ 0.0001; Figure 4D).

In summary, RBCs in the degenerated retina can also be divided into two functional types that are interconvertible by membrane potential manipulation. However, in the *rd1* retina, currents of type 2 RBCs are significantly reduced compared to the wild-type retina and the linear current component clearly prevails.

### Origin of the Strong Rectifying Outward Current in Type 2 RBCs

Since the rectifying outward current is a trademark of type 2 RBCs in the healthy retina that diminishes in the degenerating retina, we next set out to investigate its origin. An obvious candidate is the BK channel. BK channels are high-conductance potassium channels activated by both, depolarization (≥50 mV) and nanomolar increases in local calcium (Barrett et al., 1982; Yang et al., 2015). In the rodent retina, BK channels have been described on horizontal cells where they are involved in the tuning of the membrane potential (Sun et al., 2017) and on A17 amacrine cell processes forming close contacts to RBCs terminals where they modulate the release of GABA (Grimes et al., 2009; Tanimoto et al., 2012). However, BK channels have not yet been anatomically verified on murine OBCs.

**Figure 5:**
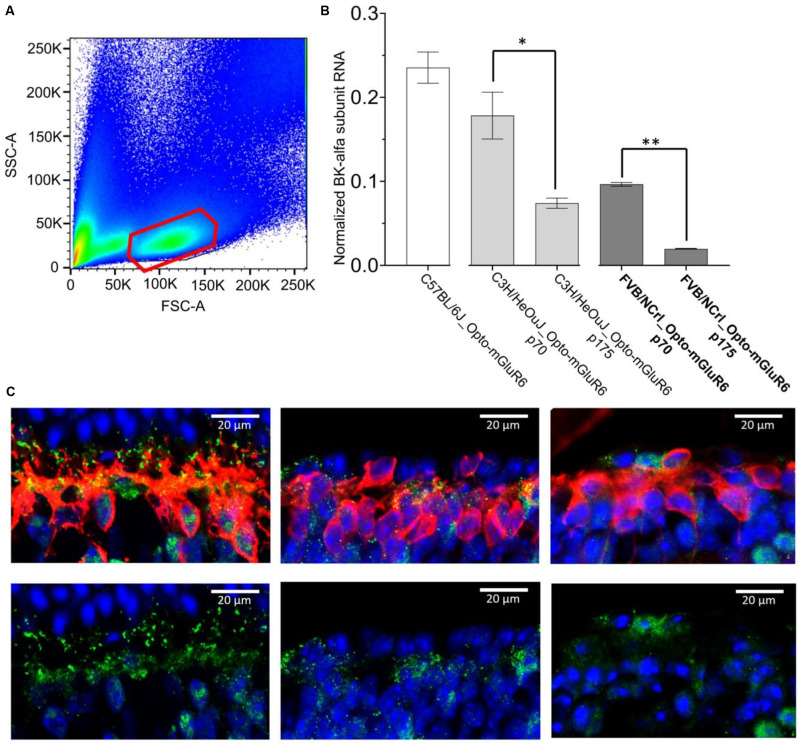
BK channel expression in RBCs is drastically reduced in degeneration. **(A)** FACS sorting of red-fluorescing (TuboFP635) OBCs. The figure represents an illustrative FACS scatter plot, with positive (red) and negative cell populations from a pool of 14 dissociated retinas. **(B)** BK channel RNA levels in OBCs from *C57BL/6J_Opto-mGluR6* mice (p 175), *FVB/NCrl_Opto-mGluR6* mice (p70 and p 175), **p = 0.007 and *C3H3/HeOuJ_Opto-mGluR6* mice (p70 and p 175), (*p = 0.02). **(C)** anti-BK immunolabeling (green) on retinal cryosection of *C57BL/6J_Opto-mGluR6* mice (left), *FVB/NCrl_Opto-mGluR6* mice at p70 (middle) and at p 175 (right). RBC were identified by anti-PKCα immunolabeling (red). Images were taken as single optical sections (770 nm) on a Zeiss LSM880 confocal microscope (63×, NA: 1.4).

We determined BK RNA and protein levels from OBC extracts of *C57BL/6J* and *rd1* retinas. For quantitative PCR we extracted RNA from sorted red-fluorescing OBCs (Figure 5A) of 14 retinas each from transgenic *FVB/NCrl_Opto-mGluR6*, *C3H/HeOuJ_Opto-mGluR6*, and *C57BL/6J_Opto-mGluR6* mice. We tested two time points (p70 and p175) in the two *rd1* strains with different degeneration timelines, *FVB/NCrl* as a rapidly degenerating model and *C3H/HeOuJ* as a slower degenerating model (van Wyk et al., 2015b). As shown in Figure 5B, BK channel RNA is present in OBCs (*C57BL/6J*: 23.53% ± 1.86%) but decreases rapidly and significantly with progressing degeneration, in the slower degenerating *C3H/HeOuJ* mouse line less rapidly (p70: 17.83% ± 2.79, p175: 7.41% ± 0.6, *p* = 0.02) than in the fast degenerating *FVB/NCrl* line (p70: 9.67% ± 2.18, p175: 1.97% ± 0.02, *p* = 0.007). We corroborated our PCR data by immunolabeling on retinal cryosections. The BK channel α-subunit was identified as puncta on the RBC dendrites and somata of *C57BL/6J_Opto-mGluR6* retinas (Figures 5C,a). In the degenerating retina, the BK channel immunolabeling re-located to the RBC cell bodies concomitant with dendritic retraction (Figures 5C,b) until it completely disappeared (Figures 5C,c).

**Figure 6:**
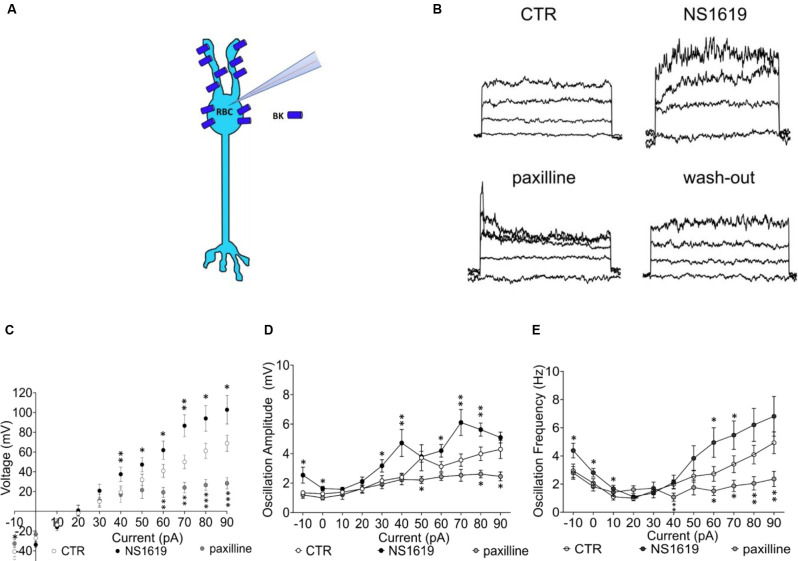
Functional BK channel characterization in the *C57BL/6J* retina. **(A)** Graphical representation of BK localization in the RBC of a healthy retina. **(B)** Raw traces of RBC current clamp recordings upon sequential application of drug, from the left: control condition, NS1619 30 μM, paxilline 5 μM, paxilline wash-out. Also see Supplementary Figure 1 that shows full reversibility of agonist and antagonist application on currents with washout. **(C)** Average RBC membrane potential in control condition (*n* = 24, CTR) and with sequential application of the BK agonist NS1619 (30 μM, *n* = 13) and the BK antagonist paxilline (5 μM, *n* = 11; *p ≤ 0.05, **p ≤ 0.02, ***p ≤ 0.001). **(D)** Average values of membrane potenital oscillation amplitude in control condition (CTR, *n* = 24), with application of the agonist (NS1619, *n* = 13) and application of the antagonist (paxilline, *n* = 11). **(E)** Average oscillation frequency in control condition (*n* = 24, CTR), with application of the agonist (*n* = 13) and application of the antagonist (*n* = 11). *p ≤ 0.05, **p ≤ 0.02, ***p ≤ 0.001.

To also functionally confirm the presence of BK channels on RBCs (Figure 6A), we measured membrane potential changes in type 2 RBCs of *C57BL/6J* retinas to current steps (10 pA, −10 pA to 90 pA) in the absence and presence of the BK channel agonist NS1619 (30 μM) and the specific antagonist paxilline (5 μM; Figure 6B, Supplementary Figure 1). The addition of the agonist increased the rectifying current at positive potentials and hyperpolarized the membrane potential by approximately 13 mV (Em control: −40.6 ± 5.3 mV, Em NS1619: −54.0 ± 5.4 mV; Figure 6C). On the contrary, the addition of the antagonist removed the rectifying current altogether and depolarized the membrane potential by approximately 8 mV (Em paxilline −32.7 ± 3.9 mV, *p* ≤ 0.05). In order to test if BK channels in RBCs are involved in membrane potential tuning, which seems to be fundamental for the categorization of an RBC into a functional type 1 or type 2, we also investigated the membrane potential oscillation amplitudes in the presence of NS1619 and paxilline (Figure 6D *p* ≤ 0.05*, *p* ≤ 0.02**, *p* ≤0.001***). NS1619 increased the oscillation amplitude and lowered the potential threshold for the generation of oscillations. Paxilline had the opposite effect, with an increased potential threshold for membrane oscillations and decreased amplitudes (Figure 6D). NS1619 also increased the oscillation frequency, whereas paxilline had the opposite effect (Figure 6E). This confirms the regulatory control of BK channels on the membrane potential, regulating the depolarization threshold as well as the oscillation amplitude and frequency.

The above results together confirm for the first time that BK channels are expressed and functional in murine RBCs. The presence of BK channel RNA in non- and early degenerated retinas and its disappearance with progressing degeneration matches our electrophysiological observation of the cessation of the strong outward rectifying current with progressing degeneration. This provides evidence that BK channel activity underlies the trademark property of type 2 RBCs.

### BK Channel Activity Is Lost in RBCs of the Degenerated Retina

**Figure 7:**
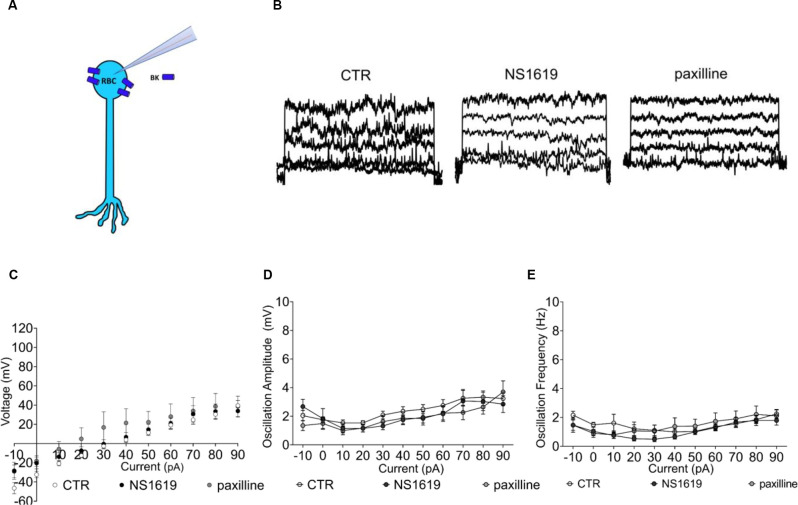
Functional BK channel characterization in the *FVB/NCrl_Opto-mGluR6* retina. **(A)** Graphical representation of BK localization in the RBC of a degenerated retina. **(B)** Raw traces of RBC current clamp recordings from the left: control condition, NS1619 30 μM, paxilline 5 μM. **(C)** Average RBC membrane potential in control condition (*n* = 31, CTR) and with sequential application of the BK agonist NS1619 (*n* = 11, 30 μM) and the BK antagonist paxilline (*n* = 8, 5 μM). **(D)** Average values of membrane potenital oscillation amplitude in control condition (CTR, *n* = 31), with application of the agonist (NS1619, *n* = 11) and application of the antagonist (paxilline, *n* = 8). **(E)** Average oscillation frequency in control condition (*n* = 31, CTR), with application of the agonist (*n* = 11) and application of the antagonist (*n* = 11).

According to the qPCR and the immunohistochemical data, BK channel expression is first re-localized to the soma and then gets progressively lost with degeneration (Figure 7A). We next tested if the few remaining BK channels in RBCs of the late degenerated *FVB/NCrl_Opto-mGluR6* (p175) *rd1* retina retained any function by applying the agonist NS1619 and the antagonist paxilline (Figure 7B). Neither the agonist nor the antagonist had any effect on the currents in RBCs of the degenerated *FVB/NCrl_Opto-mGluR6* retina, indicative of a lack or malfunctioning of BK channels (Figure 7C). Similarly, neither paxilline nor NS1619 had any effect on membrane potential oscillation amplitude or frequency (Figures 7D,E).

In summary, BK channels expressed in RBC somata of the degenerated *rd1* retina have lost their capability to regulate the membrane potential. Consequently, the membrane potential of type 2 RBCs in the degenerated retina is more depolarized and RBCs lose their high-pass filtering capacity. This may in part explain the relatively sluggish RBC responses recorded in optogentetically treated *rd1* retinas (Cehajic-Kapetanovic et al., 2015; van Wyk et al., 2015a).

## Discussion

The inner retinal layers, including the BCs, survive for an extended period after photoreceptor loss (Strettoi and Pignatelli, 2000; Chang et al., 2002), which renders BCs promising therapeutic targets for optogenetic light sensitization. Transcriptomic studies hint towards good functional preservation of BCs in the degenerated retina (Gilhooley et al., 2021), which is corroborated by multiple optogenetic studies restoring light sensitivity and basic vision in *rd1* mice (Lagali et al., 2008; Cehajic-Kapetanovic et al., 2015; van Wyk et al., 2015a) and in the first human patient (Sahel et al., 2021). Nevertheless, pathophysiological changes in BCs after photoreceptor loss were never studied directly. Insights into such changes would aid in designing the most effective strategies to restore vision.

In this study we investigated RBC function in healthy *C57BL/6J* mice and in two *rd1* mouse lines, *FVB/NCrl* and *C3H/HeOuJ*, that suffer from faster and slower photoreceptor degeneration, respectively (van Wyk et al., 2015b). Both mouse lines expressed the optogenetic actuator Opto-mGluR6 and the fluorescent reporter TurboFP635 behind the mGluR6 promoter. We identified two functional RBC types within the same morphological RBC class, which differed solely in their resting membrane potential and I/V relationships. Type 1 RBCs possess a “belly-shaped” I/V relationship and a resting membrane potential of approximately −45 mV. Type 2 RBCs have a more linear I/V relationship with an outward rectification at voltages above +50 mV and a more depolarized membrane potential of approximately −20 mV. We identified the resting membrane potential to be the toggle switch for functionality, e.g., if an RBC was of functional type 1 or type 2. In fact, type 1 was convertible into type 2 (referred to as type 2*) simply by clamping the resting membrane potential at −20 mV. This held true for the healthy and the degenerated retina. Ectopic expression of Opto-mGluR6 had no effect on the passive membrane properties of type 1 or type 2 RBCs.

Despite being grouped into one morphological cell type, some anatomical differences have been described amongst RBCs, differentiating RBC1 from RBC2 type cells (Tsukamoto and Omi, 2017). RBC1 were defined as terminating upon or within the RGC layer, whilst RBC2 were defined as terminating beyond the RGC layer. However, since half of RBCs could not be allocated to either the RBC1 or the RBC2 type, RBC1 and RBC2 were finally re-considered as a single morphological class.

The presence of the two functional cell types also in the degenerated *rd1* retina suggests the general functional preservation of RBCs in degenerated tissue. Although the ratios of functional type 1 *vs*. type 2 RBCs were similar in the healthy and degenerated retina, type 1 RBCs were even more hyperpolarized (−47.71 ± 2.70 mV) in the degenerated compared to the healthy retina (−43.48 ± 1.44 mV) and type 2 RBCs more depolarized in the degenerated retina (−19.55 ± 3.15 mV) compared to the healthy retina (−30.09 ± 2.78 mV; *p* = 0.05). The co-existence of hyperpolarized type 1 RBCs and depolarized type 2 RBCs may form the basis of contradictory reports of membrane potential changes in RBCs of the degenerated *rd1* retina. While some studies claim RBCs to be more depolarized in the *rd1* retina (Trenholm et al., 2012; Choi et al., 2014; Menzler et al., 2014) compared to RBCs of the wild-type retina, potentially mapping onto functional type 2 RBCs. Others report RBCs in the *rd1* retina to be more hyperpolarized (Borowska et al., 2011; Laprell et al., 2017), potentially mapping onto functional type 1 RBCs.

Since we performed recordings in slices from *C57BL/6J* retinas, while we used whole-mounts for *FVB/NCrl* retinas, we checked if slicing had an effect on the measured I/V curves due to potential disruption of connections onto RBCs. We recorded from slices of *FVB/Crl* retinas (Supplementary Figures 2A,B) and from isolated RBCs of *C57BL/6J* retinas (Supplementary Figures 2C–E) and found no difference in the passive membrane properties of RBCs in all three preparations. We, therefore, consider the slice and whole-mount preparations used in this study physiologically comparable, which were selected to achieve optimal access to the RBCs while keeping the tissue as intact as possible; photoreceptor degeneration renders RBCs directly accessible to the patch pipette in a retinal whole-mount, making precarious sectioning of a degenerated *rd1* retina unnecessary. Interestingly, recordings from isolated RBCs were also comparable, pointing towards a minor role of afferent connections.

We found the loss of rectification in type 2 RBCs in the degenerated retina to be due to significantly decreased expression of BK channels. BK channels are large-conductance calcium-activated potassium channels that have been described in different retinal cells, including amphibian photoreceptors (Pelucchi et al., 2008), rodent horizontal (Sun et al., 2017), ganglion (Nemargut et al., 2009), and A17 amacrine cells (Grimes et al., 2009), as well as in pre-synaptic BC terminals of the goldfish retina. No direct evidence of BK channels in murine RBCs exists. However, ERG recordings on BK knock-out transgenic mouse retinas point towards a modulatory role of BK channels in the visual response of RBCs (Tanimoto et al., 2012). We here identified BK channels on RBCs dendrites and somata in the healthy retina at the RNA and protein levels. We investigated for the first time the influence of retinal degeneration on BK channel expression in RBCs. BK channel RNA expression decreased with the progression of degeneration, more rapidly in the *FVB/NCrl_rd1* line than in the *C3H/HeOu_rd1* line. These findings were substantiated at the protein level. BK expression was drastically reduced during photoreceptor degeneration. First BK channels were re-located from dendrites to RBC somata, followed by an almost complete downregulation in late degenerated tissue. We further showed that BK channel activity could no longer be pharmacologically modulated in the degenerated *rd1* retina. Pharmacologically blocking BK channels in wild-type *C57BL/6J* type 2 RBCs depolarized the membrane potential by approximately 8 mV, which explains the depolarized membrane potential of type 2 RBCs in the degenerated retina as a consequence of BK downregulation. And more, the I/V relationship of type 2 RBCs in the degenerated *FVB/NCrl_rd1* retina resembles that of type 2 RBCs in a *C57BL/6J* retina under full block of BK channels and GABA receptors (Supplementary Figure 3). These results suggest that in type 2 RBCs of the degenerated retina not only BK channel expression is downregulated, but that also inputs from other neuronal classes are affected, such as GABAergic input from horizontal and A17 amacrine cells (Sun et al., 2017).

What is the function of BK channels in retinal cells and in particular in the RBCs? In amphibian rods, BK channels support single photon detection (Pelucchi et al., 2008) and glutamate release at the terminals (Xu and Slaughter, 2005). In the murine retina, BK channels are known to limit GABA release from A17 amacrine cells onto RBC axon terminals, which regulate excitatory synaptic transmission. Our study suggests a membrane potential tuning role of BK channels expressed in RBCs. BK channels co-localize with TRPM1 channels in the healthy and in the degenerated retina (Xu et al., 2012; Supplementary Figure 4). TRPM1 channels are non-selective cation channels triggered by the light-induced activation of OBCs. TRPM1 locally increases intracellular calcium (Martemyanov and Sampath, 2017), which could activate BK channels to repolarize the membrane potential. BK channels in RBCs may therefore have a similar role to BK channels in horizontal cells, where they were shown to keep the membrane potential in the physiological operating range, limiting cellular calcium overload and gating excitatory input (Sun et al., 2017). The depolarized membrane potential of type 1 RBCs in the degenerated *rd1* retina as a consequence of BK channel downregulation may support previously described oscillatory activity in the AII amacrine–ON cone bipolar cell network (Trenholm et al., 2012) since RBCs give excitatory input to AII amacrine cells (Wässle, 2004). Further, the loss of BK channels diminishes high-pass filtering in RBCs, which may in part explain the relatively sluggish RBC responses recorded in optogenetically treated *rd1* retinas (Cehajic-Kapetanovic et al., 2015; van Wyk et al., 2015a).

And what is the prominent current in type 1 RBCs? Type 1 and type 2 RBCs are the same anatomical cell type expressing the same complement of channels. Why does the I/V relationship then look so different? Type 1 and type 2 RBCs differ primarily in their resting membrane potential, with the membrane potential of type 1 RBCs (−43.48 ± 1.44 mV) being more hyperpolarized compared to type 2 RBCs (−30.09 ± 2.78 mV). We, therefore, focused on potassium channels that activate between −43 mV and −30 mV that could potentially mask the BK conductance in type 1 RBCs. The Kv1.3 channel recovers from inactivation at −40 mV and we, therefore, probed type 1 RBCs with the specific Kv1.3 antagonist Psora-4 (Supplementary Figure 5). We found that the prominent current in type 1 RBCs is carried by the Kv1.3 channel, which is inactivated at the more depolarized membrane potential of type 2 RBCs, rendering the BK current the dominant current to shape the I/V response.

Together, a better understanding of the cellular adaptations of OBCs during retinal degeneration will provide new avenues for future treatment strategies of blindness.

## Data Availability Statement

The raw data supporting the conclusions of this article will be made available by the authors, without undue reservation.

## Ethics Statement

The animal study was reviewed and approved by The animal research committee of Bern (approval number BE99/19).

## Author Contributions

GS conceived and performed the experiments, analyzed the data, and wrote the draft version of the article. SK conceived, supervised and funded the study, and wrote the article.

## Conflict of Interest

The authors declare that the research was conducted in the absence of any commercial or financial relationships that could be construed as a potential conflict of interest.

## Publisher’s Note

All claims expressed in this article are solely those of the authors and do not necessarily represent those of their affiliated organizations, or those of the publisher, the editors and the reviewers. Any product that may be evaluated in this article, or claim that may be made by its manufacturer, is not guaranteed or endorsed by the publisher.
