# Supplementary material for: Two Functional Classes of Rod Bipolar Cells in the Healthy and Degenerated Optogenetically Treated Murine Retina
Source: Front Cell Neurosci. 2022 Jan 13;15:809531. doi: 10.3389/fncel.2021.809531 (PMC8793500; doi:10.3389/fncel.2021.809531)
Supplement: Supplementary file 1 [file Data_Sheet_1.PDF]

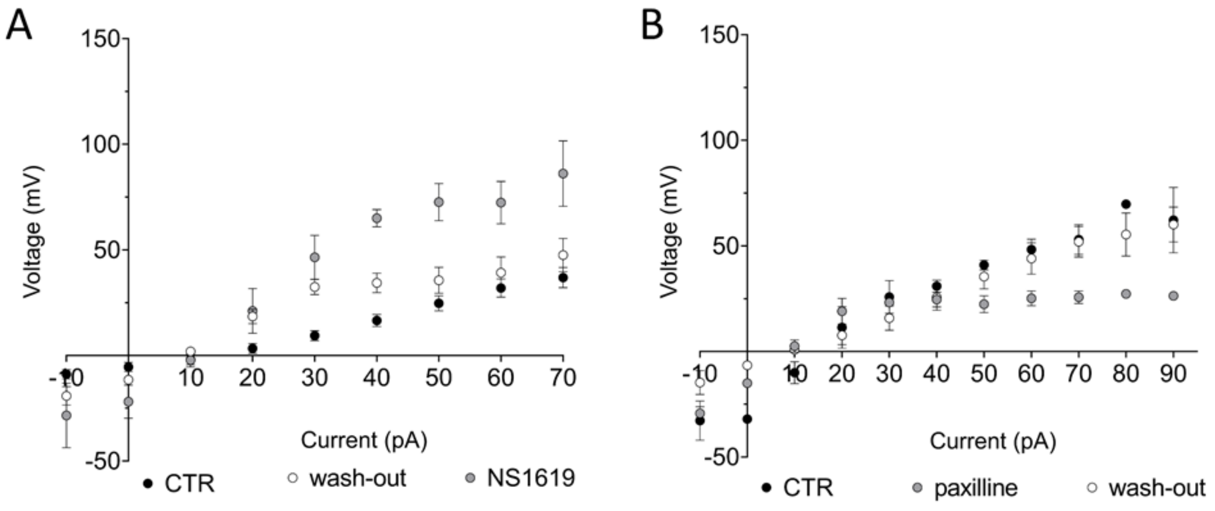

FIGURE S1 | Wash in and wash out of BK activator (NS1619) and blocker paxilline in *C57BL/6J* retinas. (A) Average RBC membrane potential in control conditions (n=3), with NS1619 (30 µM, n=3) and after wash-out of NS1619 (n =3). **(B)** Average RBC membrane potential in control conditions (n=3), with paxilline (5 µM, n=3) and after wash-out of paxilline (n =3). The drugs were applied sequentially.

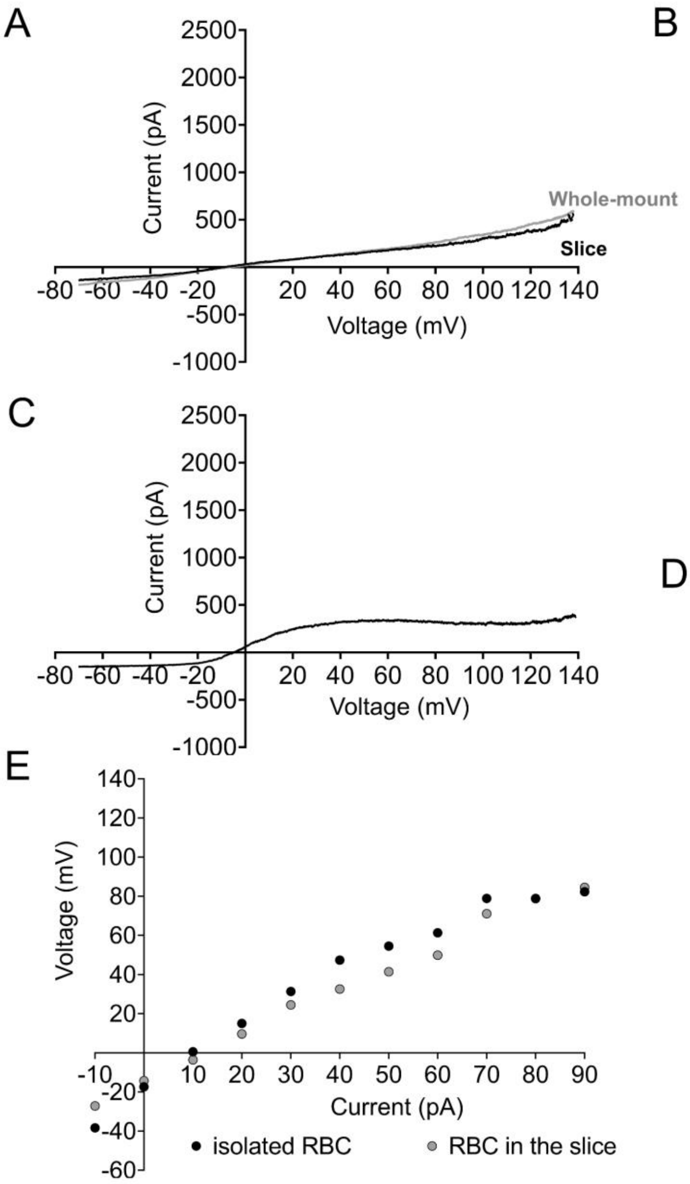

FIGURE S2 | Passive membrane properties of RBCs are maintained in electrophysiological recordings from different tissue preparations. (A) Type 2 RBC response recorded from a RBC in a *FVB/NCrl_Opto-mGluR6* retinal slice. **(B)** DIC image of the patched RBC in (A). **(C)** type 1 RBC response from isolated RBC from a *C57BL/6J* mouse retina. **(D)** Infrared image of isolated RBC taken on a Nikon Eclipse E600FN (40x, NA 0.80). **(E)** Voltage-current relationship recorded in an isolated RBC compared to a recording from a RBC in a retinal slice.

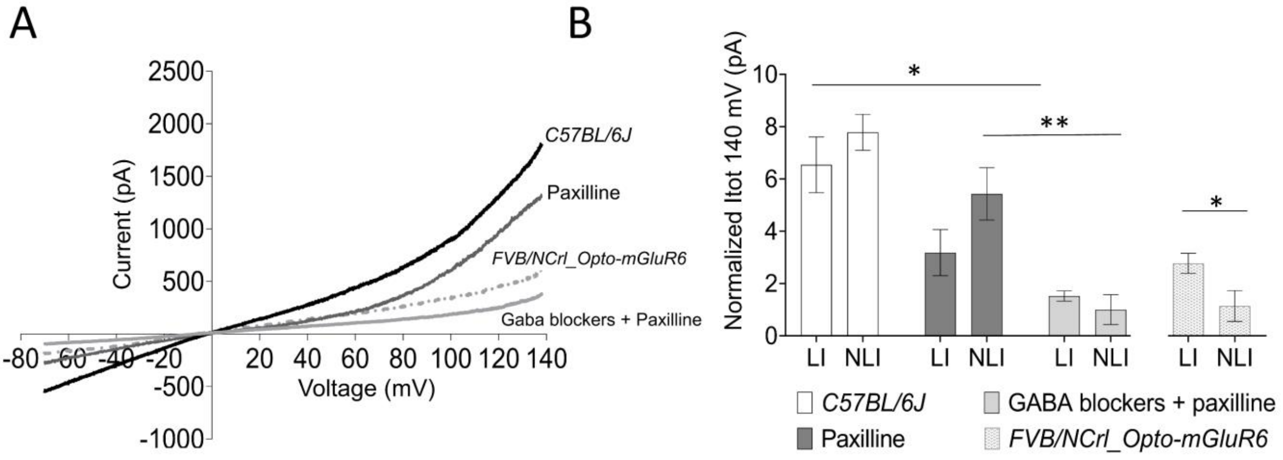

FIGURE S3 | Influence of BK channel (paxilline) and GABA channel blockers on the RBC conductivity. (A) I/V relationship of type 2 RBCs in the *C57BL/6J* retina (n = 11) with pharmacological block of BK channels alone (paxilline 5 µM, n= 6) and in combination with GABA blockers (TPMPA 50 µM; SR-95531 10 µM, n = 4) compared to type 2 RBC currents in the *FVB/NCrl_Opto-mGluR6* retina. The type 2 RBC current in the *rd1* retina resembles a type 2 RBC current in a healthy retina lacking BK channels and GABAergic input from amacrine cells. **(B)** Linear (LI) and non-linear (NLI) current components for the recordings shown in (A).

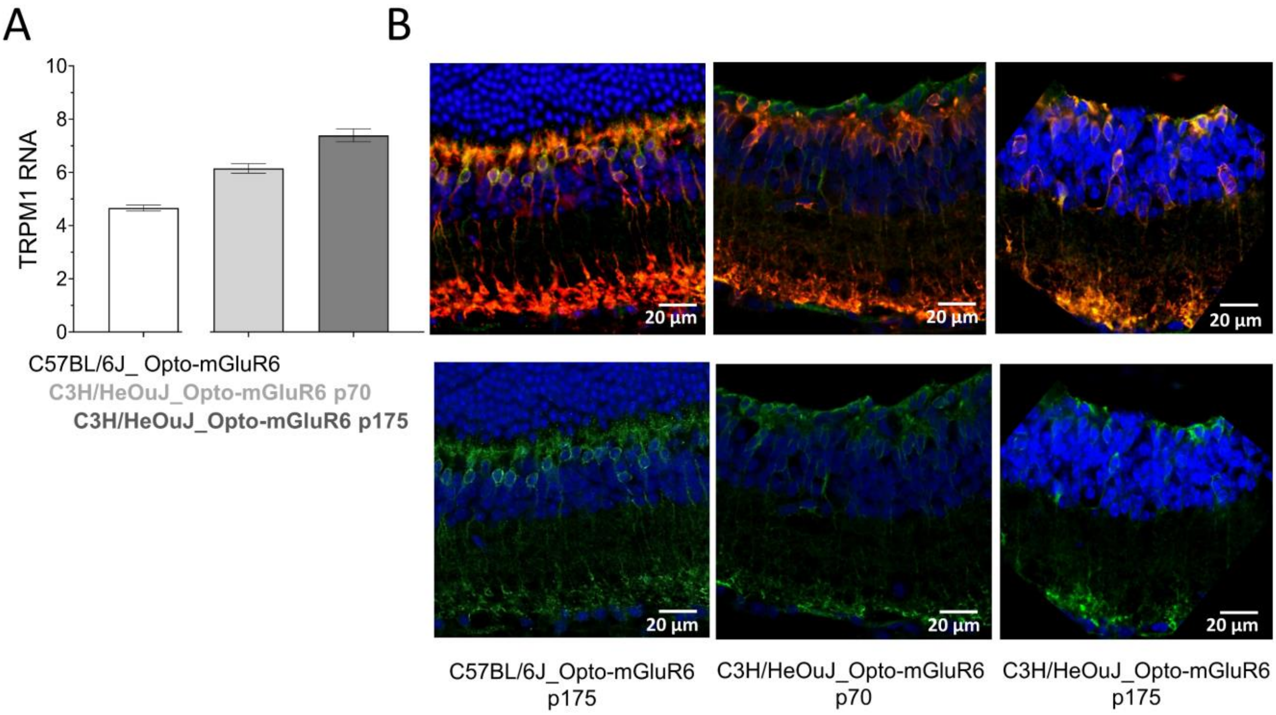

FIGURE S4 | TRPM1 channel RNA levels and protein immunolocalization on RBCs in healthy and degenerated retina. **(A)** TRPM1 RNA in *C57BL/6J_Opto-mGluR6* mice (p175) and in *C3H/HeOuJ_Opto-mGluR6* mice (p70) and (p175). **(B)** TRPM1 immunolabeling on retinal cryosection of *C57BL/6J_Opto-mGluR6* (p175) mouse retina (left), *C3H/HeOuJ_Opto-mGluR6* mouse retina at p70 (middle) and at p175 (right). TRPM1 re-localizes from the dendrites to the somata of RBCs in the degenerating retina. Green: anti-TRPM1, red: anti-PKCα (RBCs). Antibodies: rat polyclonal antibody against TRPM1 (1:100, BiCell Cat$_{\#11021}$, RRID: AB_2895222), mouse monoclonal protein kinase Cα (1:750, Invitrogen Cat$_{\#sc8393}$, RRID: AB_628142), rat monoclonal antibody Alexa 488 (1:400, Invitrogen Cat$_{\#A\text{-}11006}$, RRID: AB_ 2534074), mouse polyclonal CY3 (1:400, Invitrogen Cat$_{\#A10521}$, RRID: AB_2534030). Images were taken as single optical sections (770 nm) on a Zeiss LSM880 confocal microscope with a (63x, NA 1.4) objective.

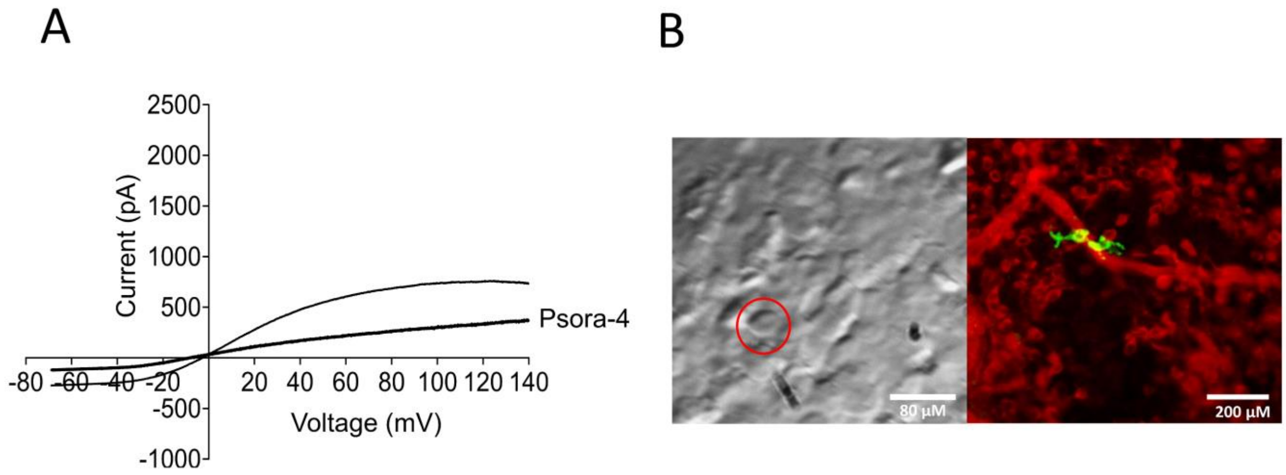

FIGURE S5 | Kv1.3 is the prominent current in type 1 "belly-shaped" RBCs of *FVB/NCrl_Opto-mGluR6* retinas. (A) I/V relationship for type 1 responses recorded from RBCs in control condition (n=6) and with Psora-4 (100nM, n=6), a specific antagonist of Kv1.3 channels. **(B)** top: live image of a RBC during the electrophysiological recording the image was taken with infrared camera GP-CAM3 Altair Astro at Nikon Eclipse E600FN (40 x, NA 0.80); bottom: photomicrograph (3µM) of an injected and immunohistochemically labelled RBC (green) as one of the TurboFP635 expressing OBCs viewed on a Zeiss Axio Vert.A1 epifluorescence microscope (40 x, NA: 0,6).
